# Visual and Rapid Detection of Porcine Epidemic Diarrhea Virus (PEDV) Using Reverse Transcription Loop-Mediated Isothermal Amplification Method

**DOI:** 10.3390/ani12192712

**Published:** 2022-10-09

**Authors:** Chunhua Li, Jieling Liang, Dan Yang, Qi Zhang, Denian Miao, Xizhong He, Yanan Du, Wanjing Zhang, Jianping Ni, Kai Zhao

**Affiliations:** 1 Institute of Animal Husbandry and Veterinary Science, Shanghai Academy of Agricultural Sciences, Shanghai 201106, China; 2 Key Laboratory of Agricultural Genetics and Breeding, Shanghai Academy of Agricultural Sciences, Shanghai 201106, China; 3 School of Life Science, Taizhou University, Taizhou 318000, China; 4 College of Life Sciences, Shanghai Normal University, Shanghai 200234, China; 5 Biotechnology Research Institute, Shanghai Academy of Agricultural Sciences, Shanghai 201106, China

**Keywords:** porcine epidemic diarrhea virus, RT-LAMP, visual detection

## Abstract

**Simple Summary:**

Porcine epidemic diarrhea (PED) is a severe disease which has led to tremendous economic losses in the swine industry all over the world. The early detection of its pathogen (PEDV) is vital to prevent and cure this disease. Here, we report the development of a new visual diagnostic test for PEDV—the reverse transcription loop-mediated isothermal amplification (RT-LAMP) method. This new assay proved to be specific and sensitive when applied to clinical specimens.

**Abstract:**

Porcine epidemic diarrhea virus (PEDV) can cause severe infectious porcine epidemic diarrhea (PED) and infect different ages of pigs, resulting in sickness and death among suckling pigs. For PEDV detection, finding an effective and rapid method is a priority. In this study, we established an effective reverse transcription loop-mediated isothermal amplification (RT-LAMP) method for PEDV detection. Three sets of primers, specific for eight different sequences of the PEDV N gene, were designed in this study. The optimized RT-LAMP amplification program was as follows: 59 min at 61.9 °C and 3 min at 80 °C. The RT-LAMP results were confirmed with the addition of SYBR Green I fluorescence dye and with the detection of a ladder-like band by conventional gel electrophoresis analysis, which demonstrated a significant agreement between the two methods. The LOD of PEDV by RT-LAMP was 0.0001 ng/μL. Compared with RT-LAMP, the traditional RT-PCR method is 100-fold less sensitive. The RT-LAMP results had no cross-reaction with porcine parvovirus (PPV), porcine circovirus type 1 (PCV1), porcine pseudorabies virus (PRV), porcine circovirus type 2 (PCV2), rotavirus (RV), transmissible gastroenteritis virus (TGEV) and porcine reproductive and respiratory syndrome virus (PRRSV). Consequently, the newly developed RT-LAMP method could provide an accurate and reliable tool for PEDV diagnosis.

## 1. Introduction

Porcine epidemic diarrhea virus (PEDV) is a crucial enteric pathogen, resulting in highly contagious porcine epidemic diarrhea (PED), along with characterized clinical symptoms, such as acute watery diarrhea, vomiting and dehydration, which cause high mortality in neonatal piglets [1]. PEDV is a positive-sense single-stranded RNA virus belonging to the family *Coronaviridae,* with a typical corona-like morphology and an average diameter of 130 nm [2,3]. The genome of PEDV is approximately 28 kb in size and contains 5′-cap structures, a 3′-poly(A) tail, and ORFs which encode 16 non-structural proteins (NSPs), four structural proteins—namely, spike (S), envelop (E), membrane (M) and nucleocapsid (N)—and an accessory protein ORF3, respectively [4,5]. There are different PEDV strains, including non-S INDEL and S INDEL strains, and some need trypsin when cultured in vitro while others do not [4].

PEDV was first discovered in England in 1971, and was isolated and identified in Belgium in 1978 [6]. Since then, PEDV has spread throughout Europe and Asia, becoming an epidemic disease in many countries [7,8,9]. In China, PEDV has been reported since the 1970s and there have been more frequent outbreaks in various swine farms since late 2010 [10,11]. PEDV first appeared in the United States in early 2013, rapidly spreading across the country and causing outbreaks in numerous pig farms, resulting in the death of more than seven million newborn piglets during one year of the epidemic [12]. Over the last 50 years, PEDV has caused severe economic losses in the swine industry and resulted in significant financial burdens on pig farms around the world. Moreover, in pig farms, PEDV was usually co-infected with other coronaviruses, such as transmissible gastroenteritis virus (TGEV) or porcine rotavirus (RV), which caused similar clinical signs and made it difficult to make a differential diagnosis. Hence, there is an urgent need to develop novel, simple and efficient methods for detecting PEDV. The rapid and accurate diagnosis of PEDV is critical for the prevention and control of the disease.

At present, many methods have been reported to detect PEDV, such as conventional virus isolation, direct electron microscopy (EM), enzyme-linked immune sorbent (ELISA) and molecular biological techniques, including reverse transcription–polymerase chain reaction (RT-PCR), in situ hybridization and real-time RT-PCR [13,14,15,16,17,18]. However, these methods are time-consuming and require highly trained personnel or expensive and complicated instruments. For example, virus isolation is a classical method to determine PEDV. However, different PEDV strains have differences in culture properties. Furthermore, PEDV is not easily adapted to cell lines and might fail to isolate the virus even if taking several weeks. So, this method does not meet the time requirements for the prevention of epidemics. The RT-PCR and real-time RT-PCR techniques require both qualified technicians and sophisticated instruments, limiting the application of these methods in the field or in poorly equipped laboratories.

A rapid and visual reverse transcription loop-mediated isothermal amplification (RT-LAMP) method is a possible solution to meet the need. LAMP is a technique that amplifies DNA specifically in vitro [19]. This method uses two or three sets of primers to identify six or eight sections of the target segment and specifically amplify them in a common water bath. The products of LAMP have a ladder-like pattern which can be visually examined after the addition of a fluorescent dye, such as SYBR Green I, and the positive LAMP reaction solution can be visually observed when it turns green, while it remains orange in the absence of amplification [20,21]. Compared with the other detection methods mentioned above, the LAMP method is a simple, rapid, sensitive and convenient method on the ground that does not need expensive or complicated equipment, except for a water bath or heating block to finish amplification [22]. The LAMP approach has successfully detected numerous pathogens, for instance, measles virus [23], porcine circovirus type 2 [24], avian influenza virus [25], porcine reproductive and respiratory syndrome virus [26], and foot-and-mouth disease virus [27].

Up to now, several methods based on LAMP have been developed for the diagnosis of PEDV [28,29,30,31,32,33]. RT-LAMP and real-time RT-LAMP for PEDV detection were established in recent years. With regard to the analysis methods of PEDV RT-LAMP results, the following detection means were adopted: agarose electrophoresis, vertical flow visualization strip and turbidity analysis by real-time turbidimeter, or DNA intercalating dye-mediated visual observation as used in previous reports [28,29,30,31,32,33]. However, vertical flow visualization strip assay, fluorescence dye detection and real-time turbidity analysis also require additional instruments, such as a visualization strip cassette, a fluorescence detector and a real-time turbidimeter, which limit the applicability of the RT-LAMP method as a field diagnostic assay. The simple and visual judgement of results is better and more suitable for detection. In the present study, primers were designed on the basis of the extremely conserved *N* gene [34,35], and a visual RT-LAMP method was established for the detection of PEDV. This method is simple, convenient, sensitive and specific in the experimental and clinical tests.

## 2. Materials and Methods

### 2.1. Viral Materials

Viral strains used for RT-LAMP assay were acquired from the Institute of Animal Husbandry and Veterinary Science, Shanghai Academy of Agricultural Sciences. PEDV (CV777 and DR13 strain), porcine circovirus type 1 (PCV1, NJ03 strain), porcine parvovirus (PPV, S-2 strain), porcine circovirus type 2 (PCV2, SH strain), porcine reproductive and respiratory syndrome virus (PRRSV, ATCC VR2332 strain), porcine pseudo rabies virus (PRV, Bartha K61 strain), rotavirus (RV, JS strain) and transmissible gastroenteritis virus (TGEV, attenuated H strain) were included and propagated in susceptible cells.

### 2.2. DNA and RNA Extraction

The total RNAs of PEDV, PRRSV, RV and TGEV were extracted from 200 μL cell culture supernatants using Blood Viral DNA/RNA kit (BIOMIGA Inc., San Diego, CA, USA), according to the manufacturer’s instructions. The total DNAs of PPV, PCV1, PCV2 and PRV were obtained by following the instructions on the kit.

### 2.3. Primer Design

Three pairs of RT-LAMP primers targeting the *N* gene of PEDV (GenBank accession number: JN825712.1) were designed by Primer Explorer 5.0. The four primers (FIP, BIP, F3 and B3) were both highly specific and used to identify six regions on the target gene. The LF and LB primers were a supernumerary pair of “loop primers” and could further accelerate the reaction. Furthermore, the F3 and B3 primers were also applied in RT-PCR and the target product was 198 bp. Their sequences and positions are shown in Table 1.

### 2.4. RT-PCR

In RT-PCR assay, a 20 μL reaction mixture contained 10 μL 2× one-step reaction mix, 0.2 μM each of F3 and B3 (Table 1), 0.4 μL enzyme mix and 1 μL RNA template using a TransScript^®^ One-Step RT-PCR SuperMix kit (TransGen Biotech Co., Ltd., Beijing, China). The reaction mixture was performed according to the following program: (1) 20 min at 50 °C, 3 min at 94 °C; (2) 35 cycles including 30 s at 94 °C, 30 s at 47 °C and 20 s at 72 °C; and (3) 10 min at 72 °C. The reaction was carried out in an Applied Biosystems 2720 thermal cycler (Applied Biosystems, Foster, USA). The products from RT-PCR assay were detected by agarose gel electrophoresis.

### 2.5. RT-LAMP

In general, RT-LAMP assay was performed in the 30 μL reaction system, which included 10× buffer, 7.5 nM dNTPs, 0.02 nM of both FIP and BIP, 0.01 nM of both F3 and B3, 0.02 nM of both LF and LB, 0.02 mM betaine (Sigma, Saint Louis, USA), 5 nM DTT, 16 U Bst DNA polymerase (Vazyme Biotech Co., Ltd., Nanjing, China) and 100 U ReverTra Ace (Toyobo Co., Ltd., Osaka, Japan). In addition to Bst DNA polymerase and ReverTra Ace, the above ingredients were added in turn. Then, 1 μg of extracted RNA was used as a template and ddH_2_O as the no-template controls (NTCs). The mixture was incubated at 95 °C for 5 min after which it was cooled on ice for 5 min and the Bst polymerase and ReverTra Ace were added last. RT-LAMP reacted in a traditional heating block.

### 2.6. Temperature Optimization

Reaction temperature for RT-LAMP was optimized from 55 °C to 65 °C for 59 min and terminated at 80 °C for 3 min. The reaction system was the same as method 2.5 mentioned above.

### 2.7. Specificity of RT-LAMP Method

In order to evaluate the specificity of PEDV detection by RT-LAMP, the DNAs (PPV, PCV1, PRV and PCV2), as well as the RNAs (PEDV, PRRSV, RV and TGEV) as sample templates, were used in RT-LAMP reaction under optimized conditions. A positive control containing PEDV DR13 and a negative control containing ddH_2_O were included.

### 2.8. Comparison of the Sensitivity between RT-LAMP and RT-PCR

To define the limit of detection (LOD) of PEDV, the PEDV RNA was diluted from 100 ng/μL to 0.0001 ng/μL as a template in RT-LAMP and RT-PCR assay. Both assays were performed three times for each concentration template and the NTC containing ddH_2_O as a template.

### 2.9. Analysis of RT-LAMP and RT-PCR Products

The amplification products of RT-LAMP and RT-PCR were detected by 2% (*w*/*v*) agarose electrophoresis and observed by staining with Goldview (SBS Genetech Co., Ltd., Shanghai, China). Additionally, the RT-LAMP products were visually observed according to their color by mixing each sample with 2 μL 1:10-diluted SYBR Green I (Thermo Fisher Scientific, Saint Louis, MO, USA). A positive sample will turn green while a negative sample will still remain orange.

### 2.10. Detection of Clinical Specimens

In total, 103 clinical specimens (including feces and intestinal samples) from newborn piglets with diarrhea were collected from three farms in Shanghai, China. Total RNAs were extracted from these samples and used as templates in the RT-PCR and RT-LAMP assay.

## 3. Results

### 3.1. Temperature Optimization of the PEDV RT-LAMP Method

**Figure 1:**
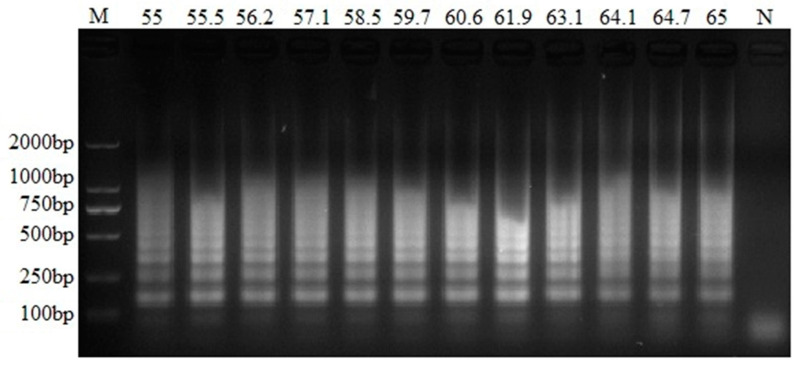
Optimal temperature of the RT-LAMP assay for PEDV. The same reaction mixtures were incubated at different temperatures (55 °C, 55.5 °C, 56.2 °C, 57.1 °C, 58.5 °C, 59.7 °C, 60.6 °C, 61.9 °C, 63.1 °C, 64.1 °C, 64.7 °C and 65 °C). The results were detected by 2% (*w*/*v*) agarose gel electrophoresis. Lane M: DL2000 DNA marker; Lane N: negative control.

The experiments showed that the optimal reaction condition for RT-LAMP was 61.9 °C (Figure 1). At this temperature, the typical ladder-like pattern is brighter and clearer than other lanes, corresponding to the highest product quantities.

### 3.2. Specificity of RT-LAMP Method

**Figure 2:**
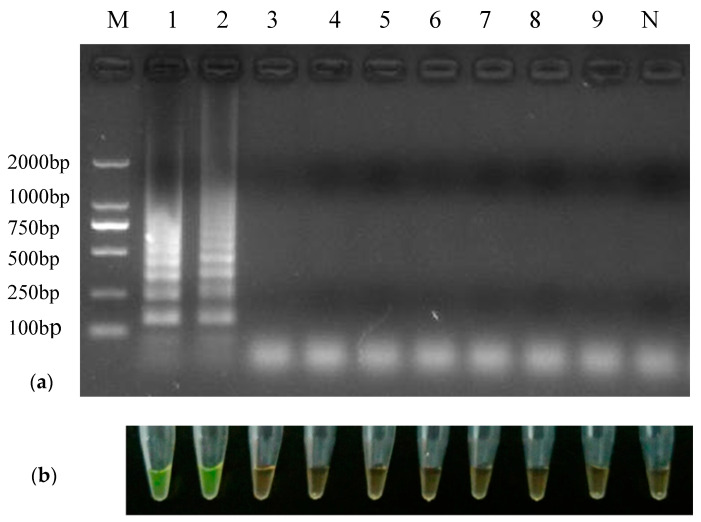
Specificity of RT-LAMP for PEDV. Amplified products of RT-LAMP were visualized by agarose gel electrophoresis (**a**) and coloration agent (**b**). Lane M: DL2000 DNA marker; Lane 1: positive control; Lanes 2–9: PEDV, PPV, PCV1, PCV2, PRV, PRRSV, RV and TEGV, respectively; Lane N: negative control.

In this study, only the RT-LAMP reaction tubes with PEDV gave typical ladder-like bands and showed a green color by using SYBR Green I, while other viruses had no bands and showed an orange color, as well as the NTC (Figure 2a,b). There were no differences between the visual observation and the gel electrophoresis analysis. These results demonstrated that the primers could be used specifically to amplify PEDV.

### 3.3. Comparison of the Sensitivity between RT-LAMP and RT-PCR

**Figure 3:**
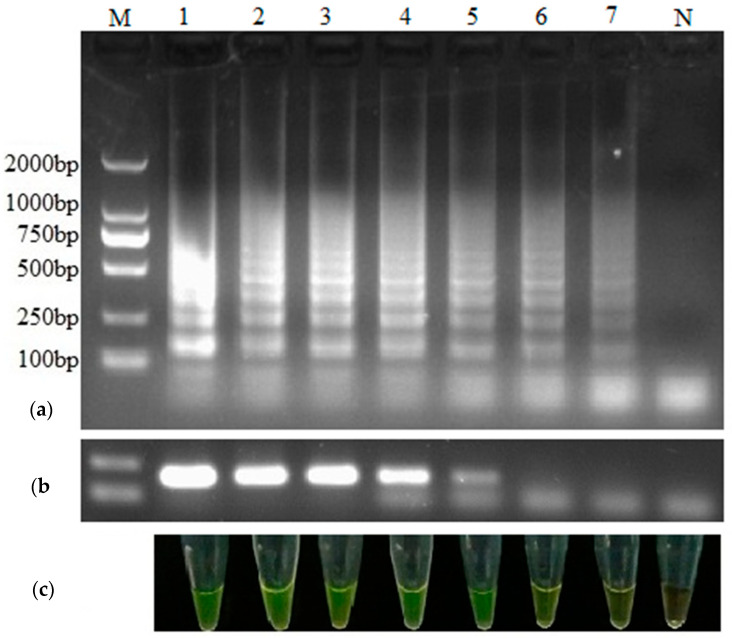
Comparison of the sensitivity between RT-LAMP and RT-PCR for PEDV. Ten-fold serial dilutions of the PEDV RNA (100 ng/μL) were used as template for RT-LAMP and conventional RT-PCR. Amplified products of RT-LAMP (**a**) and RT-PCR (**b**) were visualized by agarose gel electrophoresis and by naked eye with addition of SYBR Green I (**c**). Lane M: DL2000 DNA marker; Lanes 1–7: total RNA serially diluted 10-fold from 100 ng/μL to 0.0001 ng/μL per reaction; Lane N: negative control.

The LOD of PEDV by RT-LAMP was 0.0001 ng/μL from the result of visual observation and gel electrophoresis analysis, while the LOD by RT-PCR was 0.01 ng/μL (Figure 3). The results showed that this RT-LAMP method was 100 times more sensitive than RT-PCR assay, and the sensitivity was in line with the daily testing requirements. The length of RT-PCR was consistently 198 bp, as predicted, and its sequence was confirmed to be 100% identical to the targeted region of the PEDV *N* gene.

### 3.4. Detection of Clinical Specimens

In total, 103 clinical specimens were tested by RT-LAMP and RT-PCR methods to determine whether their sources were infected by PEDV. The results showed that 57 positive samples were by RT-LAMP and 55 were by RT-PCR, and the positive rates were 55.3% (57/103) and 53.4% (55/103), respectively (Table 2), which showed that RT-LAMP was more sensitive for PEDV detection. In summary, 54 samples were detected as positive and 45 samples as negative by both RT-LAMP and RT-PCR. The coincidence rate of these two methods was 96.1% (99/103) for clinical samples detection.

## 4. Discussion

PEDV has emerged and re-emerged in European, Asian and American countries, such as England, Belgium, Thailand, Japan, South Korea, China, the United States, Canada and Mexico, during the past 50 years, and has caused serious economic loss in the pig industry worldwide, despite the use of inactivated and attenuated vaccines to control PED outbreaks. Concerns have increasingly focused on the infectious disease. The early identification of PEDV is far more important to prevent and control the further spread of PEDV infection, as PEDV spreads rapidly and there are no effective drugs to cure the viral disease. Virus isolation is considered the standard method, but the isolation and identification of PEDV virus is complicated and time-consuming because it is not easily adapted to cell lines and cultured in vitro. While the aforementioned conventional methods have facilitated the development of PEDV detection, the high requirements of instruments and trained personnel have limited their application in the field and in poorly equipped laboratories. Therefore, the establishment of a rapid, simple and convenient method to test PEDV is extremely necessary.

Recently, the LAMP technique has become a valuable tool for the detection of various pathogens because of its high specificity, sensitivity, simplicity, rapidity and applicability in the field. Nowadays, several RT-LAMP methods have been established for RNA viruses, such as avian influenza A virus [25], porcine reproductive and respiratory syndrome virus [26], and foot-and-mouth disease virus [27], and these methods could diagnose these infectious diseases rapidly even in the field. The RT-LAMP reaction can work under isothermal conditions in a relatively short time, without the extra need for specific apparatus, such as a PCR thermocycler. The total time of RT-LAMP reaction was no more than 60 min, which is shorter than that of ELISA and RT-PCR, and it did not need either intricate pretreatment or professional staff. Hence, the RT-LAMP method has the advantages of easy manipulation and easy popularization.

In previously reported PEDV RT-LAMP assays, primers were designed to either target the N or M genes because they are more conservative than other regions of the PEDV genome. In this study, we compared sequences of the N gene and chose the most conservative region to design the primer sets in order to ensure their specificity to PEDV strains. The classical LAMP reaction was based on four primers, including two inner primers (FIP and BIP) and two outer primers (F3 and B3), which recognized six distinct regions in the target DNA [36]. The loop primers (LF and LB) were subsequently introduced to accelerate the DNA amplification, shorten the reaction time and improve the sensitivity of LAMP assay [37]. Here, we applied three sets of primers, including loop primers, to recognize the target N gene and established an optimized RT-LAMP method to visualize and detect PEDV rapidly, which was more sensitive, cost-efficient and easily manipulated. We investigated the optimized RT-LAMP method and observed that it had a high specificity for PEDV, showing no amplification products for other viruses. Betaine was added to the RT-LAMP reaction system so as to increase the rate of melting. The results demonstrate that these primers are specific and ideal for PEDV detection. Some previously reported PEDV RT-LAMP assays were established with four basic primers and without loop primers [29,30,31]. A recent report verified that the LOD of the visual detection method (vRT-LAMP) with loop primers for PEDV was at least 100-fold lower than that of vRT-LAMP without loop primers and was comparable to that of real-time RT-PCR [33].

The LOD of this RT-LAMP for PEDV sample analysis by observing and analyzing gel electrophoresis was 0.0001 ng/μL, which was lower than that of the RT-PCR (0.01 ng/μL), which was using only one set of primers. It suggested that the sensitivity of RT-LAMP is about 100-fold higher than that of RT-PCR, and visual observation can be a replacement for agarose gel electrophoresis when analyzing RT-LAMP products. In the process of evaluating clinical samples, 103 samples were analyzed by RT-LAMP and RT-PCR, and the detection rates were 55.3% (57/103) and 53.4% (55/103), respectively. The consequence of clinical samples also demonstrated that the sensitivity of RT-LAMP was higher than that of RT-PCR, which was in line with previous results [28,32]. Compared with RT-PCR, the detection rate of ELISA was much lower in the same detection conditions [38,39]. Hence, the detection rate of RT-LAMP is much higher than conventional detection methods, indicating that RT-LAMP has a high sensitivity.

For visual observation, we selected the SYBR Green I dye, which showed green (positive) or orange (negative), instead of other dyes, such as Picogreen dye, calcein and hydroxynaphthol blue, to replace the traditional gel electrophoresis analysis. In real-time RT-LAMP for PEDV, which needs an LA-320C loopamp real-time turbidimeter to measure the turbidity, the results showed that there were no differences observed in the sensitivity between the real-time turbidity and visual fluorescence detections that were associated with the real-time LAMP assay [30]. The detection limit of RT-LAMP combined with a vertical flow visualization strip (RT-LAMP-VF) was 10 pg [31]. The LOD of PEDV in our RT-LAMP assay was 0.0001 ng/μL which is more sensitive than that of RT-LAMP-VF. Our RT-LAMP can realize visual detection by adding SYBR Green I after the reaction which could avoid a false positive without incubating dye with the reagent. Therefore, the visual RT-LAMP for detecting PEDV is a good choice for less-equipped laboratories and fields. Here, we complemented and extended the superior methods for PEDV detection, and also provided an alternative option. Although there were concerns that opening the lid of the reaction tube and adding the fluorescence dye would risk contamination, this did not occur if the surgical procedures were well followed. In general, some precautions should be taken to prevent the occurrence of false positive results or contaminations in order to obtain a reliable RT-LAMP test. For example, the use of separate operating areas and aerosol-resistant pipette tips should be adopted. Meanwhile, used pipette tips and reaction vessels should be collected in airtight containers. Furthermore, dividing the reagents into aliquots and finishing the negative control first is a better way to avoid contamination.

## 5. Conclusions

In this study, we established a simple, rapid and sensitive RT-LAMP method for the on-site and visual detection of PEDV. This method can be performed in a water bath or heating block under isothermal conditions (61.9 °C) within a short time (59 min) and without PCR equipment. The LOD of this visual RT-PCR is 0.0001 ng/μL, which meets the requirement for the detection of clinical samples. Hence, this method is effective and convenient, and provides an alternative for detecting PEDV in the field and in less-equipped laboratories.

## Figures and Tables

**Table 1:** Sequences of primers used for RT-LAMP and RT-PCR analysis.

Primer	Type	Position (nt) ^1^	Sequence (5′-3′)
PEDV F3	forward outer primer	842–859	GGAGGAGAATTCCCAAGG
PEDV B3	reverse outer primer	1022–1039	AAGAGTCCGCTAGCTCAC
PEDV FIP	forward inner primer (F2-F1c)	F1c: 900–924 F2: 860–877	TTCCGCATCTCCAAAATTTTTGAAG GCGAAAATAGCGTAGCAG
PEDV BIP	reverse inner primer (B1c-B2)	B1c: 939–959 B2: 1002–1019	TGTTGATGCCTCAGGCTATGC ACAGCCACATTACCACCA
PEDV LF	Loop forward primer	879–895	CCCTGGGTCCGAAGCAA
PEDV LB	Loop outer primer	975–993	AGCACCAAATGTTGCAGCA

^1^ Gene position of each primer in the nucleotide sequence of PEDV *N* gene (GenBank No. JN825712.1). nt: nucleotide.

**Table 2:** Detection results of 103 clinical specimens by RT-LAMP and RT-PCR.

RT-LAMP		RT-PCR		Coincidence Rate
		+	−	
+	57	54	3	96.1% (99/103)
−	46	1	45	

+ Positive; − Negative.

## Data Availability

Not applicable.
